# A Wearable Navigation Device for Visually Impaired People Based on the Real-Time Semantic Visual SLAM System

**DOI:** 10.3390/s21041536

**Published:** 2021-02-23

**Authors:** Zhuo Chen, Xiaoming Liu, Masaru Kojima, Qiang Huang, Tatsuo Arai

**Affiliations:** 1 Key Laboratory of Biomimetic Robots and Systems, Ministry of Education, State Key Laboratory of Intelligent Control and Decision of Complex System, Beijing Advanced Innovation Center for Intelligent Robots and Systems, and School of Mechatronical Engineering, Beijing Institute of Technology, Beijing 100081, China; 3220190105@bit.edu.cn (Z.C.); qhuang@bit.edu.cn (Q.H.); tarai118@jcom.zaq.ne.jp (T.A.); 2 Department of Materials Engineering Science, Osaka University, Osaka 560-8531, Japan; kojima@cheng.es.osaka-u.ac.jp; 3 Global Alliance Laboratory, The University of Electro-Communications, Tokyo 182-8585, Japan

**Keywords:** wearable device, semantic segmentation, SLAM, assistance for visually impaired people, localization, semantic map

## Abstract

Wearable auxiliary devices for visually impaired people are highly attractive research topics. Although many proposed wearable navigation devices can assist visually impaired people in obstacle avoidance and navigation, these devices cannot feedback detailed information about the obstacles or help the visually impaired understand the environment. In this paper, we proposed a wearable navigation device for the visually impaired by integrating the semantic visual SLAM (Simultaneous Localization And Mapping) and the newly launched powerful mobile computing platform. This system uses an Image-Depth (RGB-D) camera based on structured light as the sensor, as the control center. We also focused on the technology that combines SLAM technology with the extraction of semantic information from the environment. It ensures that the computing platform understands the surrounding environment in real-time and can feed it back to the visually impaired in the form of voice broadcast. Finally, we tested the performance of the proposed semantic visual SLAM system on this device. The results indicate that the system can run in real-time on a wearable navigation device with sufficient accuracy.

## 1. Introduction

It is an important issue in social welfare to help visually impaired people live and travel. Governments and welfare departments of various countries have issued many policies or carried out considerable infrastructure to facilitate these people, especially the visually impaired. Nevertheless, the daily lives, especially outdoor walking and traveling, of the visually impaired is still significantly limited because of their physiological and psychological factors. Thus, it is crucial to find an effective auxiliary method. The most effective navigation method for the visually impacted people at present is to train guide dogs. However, the popularization and promotion of guide dogs among the visually impaired come at the cost of high expenses, long time, and low success rate; moreover, there are no adequate laws, regulations, and assurance to keep visually impaired and the dogs from the interference of the other people or vehicles.

**Figure 1:**
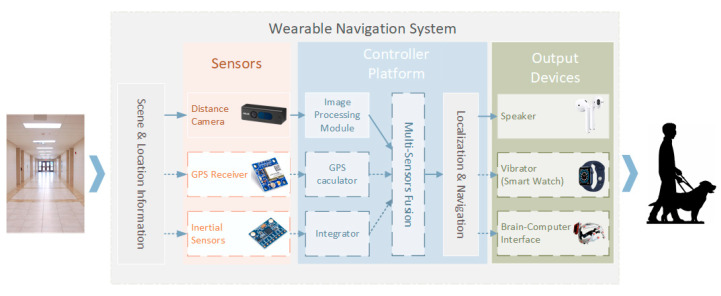
A usual wearable navigation system structure. The devices and methods we apply are shown by the solid lines.

More recently, there has been growing interest in wearable blindness-assistive devices, which have already appeared and have even been for sale. These wearable blindness-assistive devices can recognize faces, texts, traffic signals, and banknotes with high precision. As an important branch of wearable blindness-assistive devices, wearable navigation devices (WNDs) for blind people still rely on traditional methods, such as ultrasonic obstacle avoidance, GPS positioning, inertial odometry, and other indoor localization methods [1,2,3], which cannot meet the accuracy requirements of walking navigation and have significant limitation in the indoor environment. Visual navigation has become a hot research topic in recent years, which has considerable potential as WNDs. Figure 1 shows a simple WND system concept based on computer vision. GPS (Global Positioning System), images, and inertial information are collected and input into a microcomputer controller unit simultaneously. Then, positioning and navigation are performed based on the data of multi-sensor fusion among these sensors. The navigation information is sent via Bluetooth to other auxiliary devices worn by the visually impaired, such as earphones, smartwatches, and guide cane [4]. Additionally, the visual presentation-based brain-computer interface [5], which has become popular in recent years, can help visually impaired people restore their vision. It is also suitable as an output device for navigation information, especially on the WNDs that can build three-dimensional semantic scene information.

Considering that WND frequently works (especially navigates) in unknown environments, and as blind people cannot identify surroundings directly, it needs not only to determine its position, posture, and trajectory but also to establish a global map in real-time, which is precisely the same as the SLAM problem in the robotics field. The concept of SLAM was put forward in the 1980s [6] and detailed the most fundamental problem that robots need to face when moving and recognizing their position. The earliest SLAM schemes were realized by simple position sensors such as sonars and odometers, which are usually bulky in volume. According to the reports of recent researches, SLAM schemes can also be established based on vision sensors. Visual SLAM, including Monocular, Stereo, RGB-D cameras, and Lidars, has shown significant advantages in intelligent mobile robots and autonomous vehicles because of robustness, intelligence, and adaption. Some SLAM schemes, such as ORB-SLAM [7,8], LSD-SLAM [9], ElasticFusion [10], and SVO [11], are mature.

There are many significant pieces of research about the WNDs combined with SLAM. Kaiser et al. [12] described a wearable navigation system based on an a priori map established by the SLAM process of a robot. It was necessary to create a map in advance when applying it to navigation for the visually impaired people, since the early SLAM system usually relied on high-precision but heavy equipment. Lee et al. [13] proposed a method of using RGB-D cameras and some other equipment to realize real-time navigation without the need to establish a priori map through the SLAM scheme. According to their description, the program can achieve a processing speed of 28.6 Hz. However, this solution requires visually impaired people to interact with a handheld smart device, especially when setting a destination, which may bring potential inconvenience to the user. Zhang et al. [14] also used RGB-D cameras to realize navigation without prior maps. Different from the work of Lee et al., they proposed the method using voice and text recognition to determine the destination. Especially, geometric features at some specific locations are utilized to extract semantic information so that the user could follow the preset path correctly. Although Zhang et al. considered the perception of the visually impaired people during navigation, the information they provided was not clear enough. Moreover, since some other information such as the door numbers is collected by another independent camera, the user may need to wear more equipment, which would also cause some inconvenience.

The SLAM scheme applied to the WND, according to many previous studies, is different from the general SLAM scheme. The latter only needs to build a topological map and not know what objects are on the map. In the process of positioning and navigation, the WND needs to recognize various items on the map in real-time to determine whether the object is an obstacle. Therefore, a 3D semantic scene establishment solution that can extract semantic information from the map and perform localization simultaneously and in real-time is urgently required.

Much work has been done in the field of 3D semantic scene establishment. The semantic information labels were tagged by the decision-level tree random forest pixel-level semantic prediction in the early years. SLAM++, proposed by Salas-Moreno et al. [15], is the earliest prototype, which is based on the dense reconstruction of scenes and optimized by setting camera nodes and object nodes after detecting a specific object. The work of Zhang et al. [14] also proposed the idea of using semantic information to assist positioning, but they only used simple geometric features to obtain semantic information, which could not achieve 3D semantic scene recognition. However, CNN (Convolutional Neural Network) has become a popular method for image target recognition and detection with machine learning technology developed in recent years. There are many studies on CNN image semantic segmentation, such as FCN [16], DeepLab [17], ResNet [18], and PSPNet [19]. The CNN image detection and semantic segmentation are applied to autonomous driving and robotics widely. It is a considerable suit choice for us to use CNN to realize 3D semantic scene identification.

This paper has proposed a real-time 3D semantic scene SLAM solution for WNDs, integrating a semantic segmentation network and a SLAM system to construct a new semantic visual SLAM system. Firstly, we have built a wearable navigation system. We extend the data structure of map points by probability fusion between SLAM’s Mapping processing and semantic information and finally construct a SLAM system for the WNDs with semantic label output. We have done accuracy and speed evaluation using the TUM RGB-D database [20] on the wearable navigation system. This SLAM system can generate three kinds of maps, including the sparse map, dense map, and semantic map. Compared with the general semantic SLAM scheme, our work can ensure the speed of real-time operation on low-cost devices. Finally, we set up a test scenario to simulate navigation for visually impaired people through voice enlightening.

## 2. Real-Time Semantic Visual SLAM

The real-time semantic vision SLAM solution for WNDs consists of two parts: one is a high-precision SLAM system, the other is a real-time semantic segmentation network. The combination of the SLAM system and semantic segmentation network will be discussed in the third part.

### 2.1. Real-Time SLAM System

The SLAM system is the fundamental part of the Semantic visual SLAM system, which is positively correlated with the accuracy and final performance of the WND system. There are currently three methods for constructing the SLAM scheme: the first is the feature-based method, which extracts some feature points with descriptors in the image, and matches these feature points between different images for tracking and mapping; the second is the direct method, which directly calculates the luminosity changes of some pixel blocks (not only using feature points); the third is the optical flow method, which uses the optical flow changes of feature points, pixel gradient points, and even the entire picture. The semantic information needs to be fused through data association, which further requires data association and localization estimation to be decoupled. The feature-based method needs to match feature points with the current position, demonstrating compatibility with semantic information fusion.

**Figure 2:**
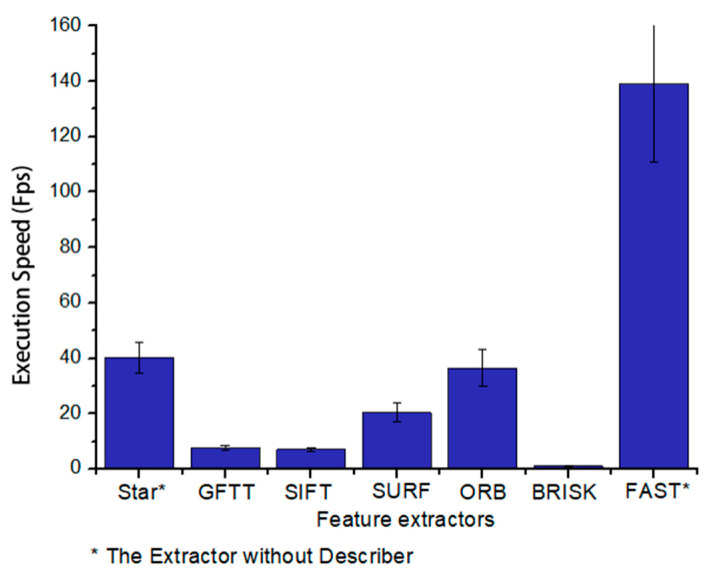
Execution speed comparison of some popular feature extractors (input image size: 640×480 , feature points < 1000).

In fact, the feature-based method is not highly efficient. As is shown in Figure 2, we have done an operation speed test with a single thread for some popular feature extractors, including Star (or CenSurE, Center Surround Extremes, by Agrawal et al. [21]), GFTT (Good Feature to Track by Shi et al. [22]), SIFT (by Lowe et al. [23]), SURF (by Bay et al. [24,25]), ORB (by Rublee et al. [26]), BRISK (by Leutenegger et al. [27]), and FAST (by Rosten et al. [28]). The feature extraction is such a time-consuming task that only a few extractors can meet real-time operation requirements. However, FAST and another extractor with better performance, Star, are both feature-only extractors without descriptors, which cannot be used directly in SLAM. Considering not only to meet the application requirements of SLAM but also to satisfy the real-time performance on the low-cost devices, we finally chose the ORB feature extractor.

It is worth noting that in recent years, a SLAM solution based on the ORB feature extractor has been proposed. As one of the most famous feature-based visual SLAM solutions, ORB-SLAM, presented by Mur-Artal et al. [7,8], has shown significant operating speed, robustness, and localizing accuracy. There are numerous designs in the ORB-SLAM to improve efficiency, which can be executed using a CPU and reach at least 30 FPS. The real-time nature of ORB-SLAM is precisely in line with the real-time requirements of this work. At the same time, it also provides interfaces for monocular, stereo, and RGB-D cameras, which provides the foundation of SLAM construction. However, the final result of the ORB-SLAM has limited readability and practicality, which needs to be processed. We first established a dense global map based on the original ORB-SLAM to ensure that the generated semantic map can match the global map. In addition, we reassigned the threads that SLAM runs and performed further processing during the extraction of keyframes to ensure the efficient operation of the entire program, which are introduced in the next section.

### 2.2. Framework of Semantic Visual SLAM

**Figure 3:**
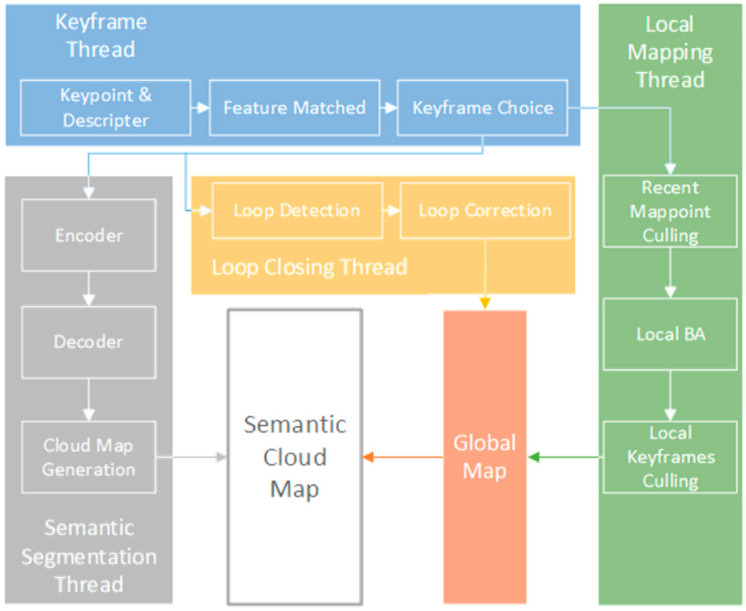
The framework of the proposed semantic visual SLAM system.

As is shown in Figure 3, the system can be divided into two parts: the SLAM end and the semantic segmentation end. The SLAM end can also be divided into three threads: Keyframe, Local Mapping, and Loop Closing. Then, the global map is constructed through global pose optimization and Loop Correction. In the meantime, semantic segmentation threads are expanded into this system. The semantic segmentation result can be mapped to a dense semantic map through the Cloud Map Generation step, which combines the generated semantic results with the depth information from the RGB-D camera and generates the corresponding position and posture information from the global map of the SLAM end.

Semantic segmentation usually takes up too much computing resource, which causes the overall system to run at a slower speed. Here, we have applied two methods in this system to solve this problem.

Maximizing the use of computing resources is a critical way to improve operational efficiency. Although localization and navigation are the essential parts of the WNDS, it is impossible to devote all computing resources to solve SLAM. Current small high-performance computing devices have begun to use GPUs to enhance AI (Artificial Intelligence) capabilities, which can usually operate in parallel. The computing ability of the GPU can be used as much as possible to handle the task of semantic segmentation, to save CPU computing resources. Due to the requirement for complex matrix operations, the semantic segmentation coincides with the functional characteristics of the GPU as well. In short, the considerate allocation of computing power by the three threads of SLAM and the semantic segmentation thread can effectively ensure the real-time performance of the system.

Secondly, many redundant frames appear in SLAM, and the semantic segmentation of these frames also causes low efficiency. Considering the step of extracting keyframes in the SLAM thread, it is feasible to record the timestamps of keyframes and then index the images near the time stamps for semantic segmentation. The keyframe refers to the graph node captured during the mapping process associated with the previous keyframe, which, at the same time, has a significant difference. The cloud points mapped by the keyframes are finally combined into a point cloud map as a result. Therefore, semantic segmentation is performed on the images closest to the keyframes so that the map with semantic information can be established through point cloud reconstruction. Semantic segmentation of images near keyframes can minimize the amount of calculation and improve the efficiency of segmentation.

### 2.3. Semantic Segmentation

Semantic segmentation by the decision-level tree random forests has many limitations on the application. With the rapid development of CNN, Neural Networks are gradually becoming more popular in the field of the pixel-level segment. Moreover, the development of the Semantic Segment also aims to apply intelligent mobile robots and automatic driving vehicles. We can choose the base segment network from existing research and improve it. A highly efficient but succinct image semantic segmentation network is needed for the parallel process between SLAM and segment network. The target detection network can replace pixel-level semantic segmentation in some schemes, but it focuses on enhancing the performance of SLAM and has little effect on generating semantic maps.

Although there are many excellent CNN image segmentation networks, most of them rely on high-performance computing equipment for training and operation. ENet (proposed by Paszke et al. [29]) is an efficient pixel-level semantic segmentation network. It uses a 34-layer residual network to form an asymmetric Encoder-Decoder structure. Due to the small number of network layers, the training speed of this network is fast, and the training model is small. To improve the accuracy and ensure the running speed, we have made a simple change of this network, mainly to increase the number of convolution layers but retain the asymmetric structure of the network.

**Figure 4:**
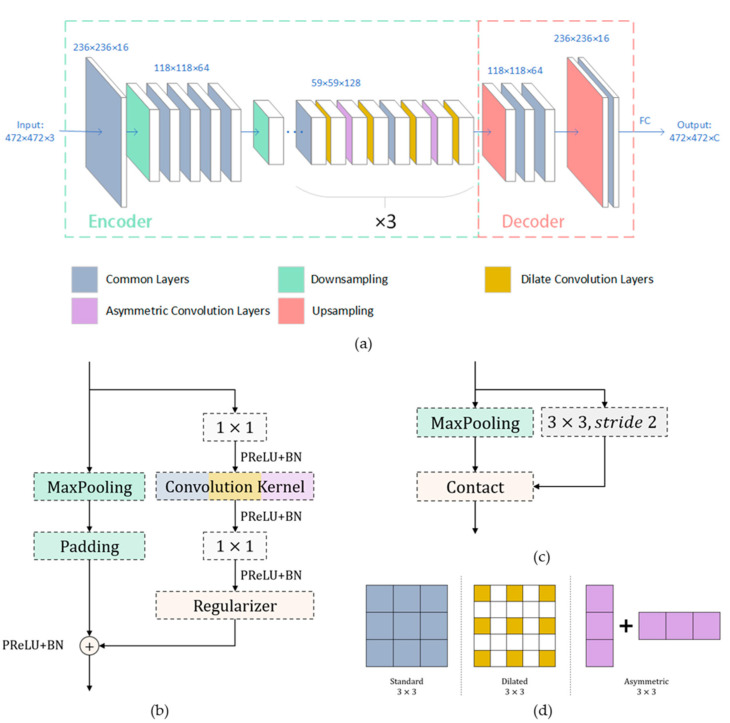
The framework of semantic segmentation network [29]. (**a**) Convolutional network main framework. (**b**) Bottleneck layers inner structure. (**c**) Initial layer. (**d**) Three kinds of convolution kernels: standard, dilated, and asymmetric kernels.

As shown in Figure 4, five kinds of convolution layers are adapted to establish the Semantic Segmentation network. The entire network can be divided into seven stages, where stages 1–5 constitute the encoder and stages 6–7 constitute the decoder. The order of the internal convolution layers of stages 3–5 is the same. Although it is not deliberately emphasized in the figure, except for the initial layer of the first stage, each layer adopts a Bottleneck structure. The Bottleneck structure is shown in Figure 4b. The initialization layer, which can be seen as Figure 4c, directly concatenates the result of convolution using a 3 × 3 (stride 2) kernel together with the max-pooling result to achieve rapid compression and reduce the storage space of a single image. There are three types of convolution kernels used in this network, including standard kernels, dilated kernels [30,31], and asymmetric kernels [31], which are shown in Figure 4d. The use of multiple convolution kernels can effectively expand the receptive field, which can significantly increase the speed and efficiency of the network while ensuring the accuracy of segmentation.

### 2.4. Probabilistic Data Association

The single semantic segmentation has not yet made the computer controller match the segmentation result with the detected terrain. Section 2.2 mentioned that the semantic information is extended to three-dimensional space based on the localization results and point cloud information, which only generates a separate semantic map. Therefore, another method is needed to associate the generated semantic map with the point cloud map, which is the so-called data association. Probabilistic Data Association was first proposed by Bowman et al. [32]. It extracts the location-related information by searching the maximum probability coincident position of the current coordinates in the point cloud map. Moreover, since the input sizes of the SLAM end and the segmentation network do not match, the two need to be processed uniformly.

When building a semantic visual SLAM system, data association is the most crucial part. In the particular scenarios applied to WNDs, this data association does not need to achieve high accuracy or correct the result of SLAM. WNDs need to perceive the semantic information of the surrounding environment in a three-dimensional manner, which means the devices need to know what they have seen and how far these objects are. Consequently, only relying on semantic segmentation of captured images cannot achieve this goal. The so-called data association refers to correlating semantic recognition results with the results of the cloud points map, which is equivalent to extending semantic information to three-dimensional space. The general SLAM solution solves the classic positioning and mapping problems. The mobile sensor moves in an unknown environment and establishes a total of M static landmark sets $L={\left\{l_{m}\right\}}_{1}^{M}$. The corresponding measurement value set of the sensor is $Z={\left\{z_{k}\right\}}_{1}^{K}$. The task of SLAM is to use the estimated landmark L to restore the sensor trajectory position and attitude set $X=\;{\left\{x_{n}\right\}}_{1}^{n}$.

(1) $$\hat{X},\hat{L}=\underset{X,L}{\mathrm{argmax}}\log p(Z|X,L)$$

The Semantic SLAM needs not only to be able to restore posture through landmarks but also to combine semantic labels with map points. Therefore, it is necessary to associate semantics signs, landmarks, and restored poses in the set $\hat{D}$:

(2) $$\hat{X},\hat{L}=\underset{X,L}{\mathrm{argmax}}\log p(Z|X,L,\;\hat{D})$$

(3) $$\hat{D}=\underset{D}{\mathrm{argmax}}p(D|X^{0},L^{0},\;Z)$$

where $X^{0},L^{0}$ are the prior estimated value and D  is the maximum likelihood estimate based on $X^{0},L^{0}$. It is worth noting that the landmarks here are those that already contain semantic information. The keyframe records the association between the restored pose X and the landmark L containing semantic information. In the process of loop closing detection, keyframes with the same semantic distribution are pre-screened as the key detection object. The semantic information will be connected with the generated point cloud image by fusing data association with landmarks. Therefore, when indexing semantic information, the keyframes that can be matched with the coordinates in the map and the corresponding semantics are extracted.

In order to make SLAM better cooperate with the semantic segmentation network, some changes in semantic information are necessary. On the one hand, the input images need to be segmented, and on the other hand, the landmarks need data expansion.

The image input size is set to 472 × 472 (pixels) to prevent the directionality problem of the images input to the network. The image input size of SLAM is the resolution of the camera 640 × 480. We unified the two by cutting. The center of these two input images coincide with each other, and the edge parts of the network input were ignored. The landmarks at the ignored positions are uniformly classified as “unclassified” to ensure matrix operations. This cutting method also avoids the mismatch, to a certain extent, that may occur when the network recognizes objects with incomplete edges on the image.

The semantic labels of landmarks are variable to avoid the classification errors that often occur. Each landmark continuously records the number of times where a keypoint is classified as a specific category during the entire SLAM process. With the work of the backend non-real-time optimization process, the largest category with a number of occurrences above a certain threshold other than uncategorized is selected as its own classification. If the mismatches are distributed with the normal distribution, this method can exclude most mismatch cases.

## 3. Experiments and Results

We have done some experimental validation of our system, especially the performance of executing speed, the experiments of Global Map, the accuracy of SLAM trajectory based on the TUM database, and the performance of Semantic Segment Network. Low-cost computers limited a large enough batch size, for which the convergence effect may not be ideal. This problem can be solved by renting a cloud server with higher performance. We also built a simple scenario to test the actual performance of the system.

### 3.1. Experimental Platform Setup

**Figure 5:**
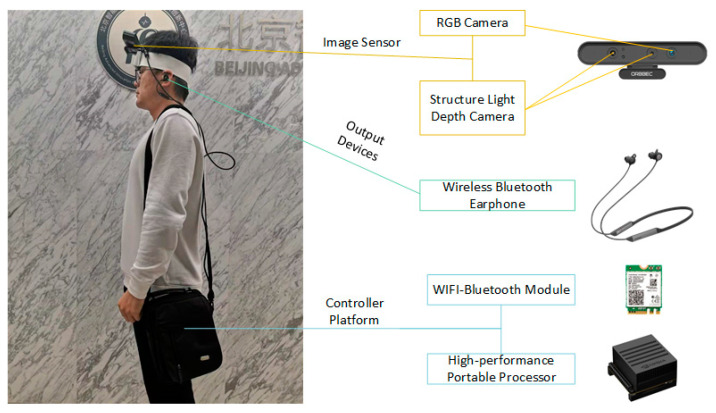
The wearable navigation system platform.

There is no doubt that the SLAM system for WNDs, which has strict requirements on the weight of the related devices and the computing ability of the controller, is different from the one used in robot navigation. Therefore, we need to reduce the hardware quantity and mass of the test platform as much as possible. The entire hardware system needs to be built on a high-performance embedded development platform. As is shown in Figure 5, we used an embedded graphics processing module made by NVIDIA as the control terminal to process the images collected by the RGB-D camera. The processed result was sent to the receiver through a WIFI-Bluetooth module with the M.2 interface. The user can obtain the output information by wearing a wireless Bluetooth earphone. The hardware part of the test system consists of only RGB-D cameras, controllers, and power supply facilities. There are no specific requirements for the wireless Bluetooth earphones so that users can purchase them according to their needs and interests. We also used the speech synthesis method provided by IFLYTEK to construct a voice broadcast solution that can be sent to the earphone through Bluetooth. Moreover, since the use of GPS and inertial odometry are effective methods for improving localization and navigation accuracy, we have retained the interface of these two devices for subsequent research.

### 3.2. Performance Evaluation of the Real-Time SLAM

We have done a variety of validations on the TUM database faced with the RGB-D camera. There are many tools of this database that we can use to give a quantitative test on the accuracy of localization and the executing speed of tracking, as Table 1 shows. The localization error of the system is about 5 cm (centimeter-level). Notably, the error of the first sample in Table 1 is huge, which is caused by the too fast running speed and severe image shaking. After the experiments, we believe that only relying on the visual odometry in SLAM is likely to produce cumulative errors under long-term working conditions. Therefore, we have achieved navigation through voice enlightening, but accurate navigation requires the solution to be combined with IMU (indoor) or GPS (outdoor) to reduce errors.

**Figure 6:**
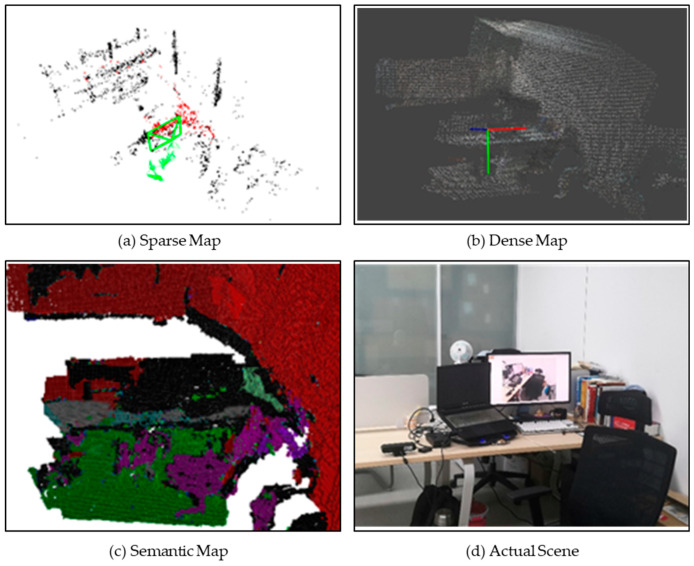
Different maps generated by the proposed semantic visual SLAM.

The single SLAM speed can reach 30 fps (limited by the specification of the camera in the database), which meets the real-time requirements completely. When the whole system is performed, the SLAM speed evaluation result can be seen in Table 2. During operation, the computing resources occupied by the graphical interface cannot be ignored. Therefore, the entire system still has considerable potential for speed improvement. We designed an experimental scene for on-site testing with the background of guiding the visually impaired into the workstation. Our work can generate sparse maps, dense maps, and semantic maps based on octrees, as shown in Figure 6. After feeling that the camera is in a static state at the same position for a period of time, the program can return the most critical semantic information in the related keyframe and the distance and direction between the centroid of the object and the camera (e.g., “chair, front-left, 0.5 m”). Utilizing the offline speech synthesis platform provided by iFLYTEK^®^, we can produce sound and send it to the earphone through the Bluetooth module.

### 3.3. Result of Semantic Segmentation

We have evaluated the Semantic Map. We also trained and validated the CNN in a low-cost computer installed with an NVIDIA GTX 1060 with 6 GB of Video Memory. The fundamental specifications are listed in Table 3. Training reaches a steady state after about 10k iterations, and the final mIoU (mean Intersection over Union) of training is about 70%. The mIoU evaluation method is as follows:

(4) $$m\mathrm{IoU}=\frac{1}{k+1}\overset{k}{\underset{i=0}{\sum }}\frac{p_{ii}}{\sum_{j=0}^{k}p_{ij}+\sum_{j=0}^{k}p_{ji}-p_{ii}}$$

where k is the number of the input test samples, p is a rate of the total that stands for the comparison output between evaluated and actual results, and footmarks, and i and j, stand for the right and wrong situation respectively. The same two footmark letters of the p refer to the right judgment. Obviously, the sum of the four different *p* is just 1.

Some well-known methods were tested on our device, of which the results can be seen in Table 4. This scheme can be executed in real-time on the device, though because there was not enough computing capacity, it reached about 13 fps. The output training model is only 10M and can be developed on embedded devices.

**Figure 7:**
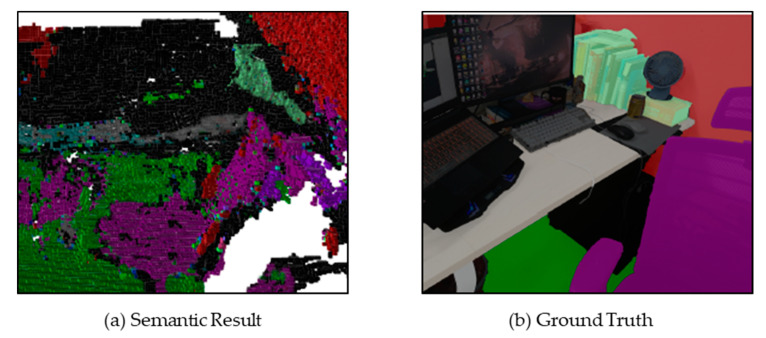
Semantic cloud map established in an Indoor Environment.

We have discussed the performance of the SLAM system and the segmentation network separately. In this part, we show the overall performance of the system. The speed of the system is lower than that of the individual test but not obvious (about 25 Fps for SLAM and about 10 Fps for Semantic Segment). Figure 7 shows the comparison between the Ground Truth of the scene part and the 3D semantic map. It can be seen that the 3D semantic map can also run normally for a complicated scene.

## 4. Conclusions

This paper has proposed a scheme to apply mature SLAM and semantic segmentation methods to wearable assistive devices for visually impaired people. We chose SLAM based on ORB feature extraction as the basis and constructed a real-time visual semantic SLAM solution for WNDs through a fast and efficient semantic segmentation network. To ensure the real-time performance of the system running on low-cost embedded devices, we made full use of the computing resources of the device and divided SLAM and semantic segmentation into multiple threads, which were allocated to different types of computing units according to their characteristics. The experimental results show that even under the condition of retaining the visual graphical interface, the program can still run on embedded devices at a processing speed of about 25 Fps. At the same time, the error is limited to centimeter-level. Finally, the generated semantic map and the SLAM cloud map are associated with each other through probabilistic association, which can further utilize mature speech synthesis solutions to broadcast location and object information to the visually impaired and further enlighten them to reach the destination. In general, the experimental results show that this system can be run in real-time on the platform we built, and the semantic map and SLAM map can be established synchronously.

Our solution shows the potential of semantic SLAM in wearable navigation devices, which can still improve in several aspects. Firstly, consider the miniaturized, lightweight, and integrated design. In fact, there are many small high-performance chips and high-precision cameras that can be used to develop wearable navigation devices. Secondly, the user experience can be improved through developing the navigation program and enhancing the human-computer interaction and appearance of the production. In addition, research in the fields of semantic SLAM fusion and multi-sensor fusion will also help further to improve the efficiency and accuracy of wearable navigation devices. Limited by the computing power and accuracy of the device, we can only implement voice-heuristic navigation currently. Accurate navigation requires the use of multi-sensor fusion, integrating GPS, IMU, and other indoor localization methods to obtain high-precision location information. Moreover, the interaction method between wearable devices and visually impaired people is also an important research topic. Simplifying the interaction operation as much as possible and increasing voice communication and text understanding can provide greater convenience for the visually impaired. In the future, we will try to utilize the GPS module and inertial measurement units while reducing the size and weight of the system to improve the operating accuracy and application scenarios of the devices.

## Figures and Tables

**Table 1:** SLAM trajectory evaluation.

Name	Trajectory Error Specifications		
	Root Mean Squared Error/m	Median/m	Max Error/m
Fr1_360	0.2411	0.2275	0.4667
Fr1_desk	0.0225	0.0150	0.0822
Fr1_floor	0.0216	0.0170	0.0656
Fr1_room	0.0303	0.0249	0.1076
Fr2_hemi	0.0954	0.0939	0.3040
Fr2_pioneer	0.0716	0.0754	0.1470
Fr3_office	0.0098	0.0092	0.0256

**Table 2:** SLAM execution speed evaluation.

Name	Speed Specification		
	Keyframes Number	Average Tracking Times/s	Frames Per Second *
Fr1_360	127	0.236929	23.45
Fr1_desk	62	0.377419	20.25
Fr1_floor	56	0.890536	27.15
Fr1_room	224	0.218304	15.4
Fr2_hemi	523	0.174914	20.85
Fr2_pioneer	373	0.195565	20.58
Fr3_office	224	0.388795	16.1

* Refers to the SLAM processing speed with the visual interface rather than the speed of the input video stream.

**Table 3:** The performance of the proposed semantic segment networks.

The Output Parameter of Semantic Segment Networks	
Output size	10.3 MB
Speed of Segment	~13 Fps
mIoU	60.2% for Validation
Pixel Accuracy	89.1% for Validation

**Table 4:** Comparison of the segmentation execution speed.

Name	Segment Speed (Fps)	mIoU for Validation (%)
FCN [16]	1.1	61.2
DeepLab V3+ [17]	0.3	85.1
ICNet [33]	8	68.5
SegNet [34]	5	53.0
Our work	13	60.2

## Data Availability

The data presented in this study are available on request from the corresponding author. The data are not publicly available due to restrictions of privacy.
